# Emerging sensing and modeling technologies for wearable and cuffless blood pressure monitoring

**DOI:** 10.1038/s41746-023-00835-6

**Published:** 2023-05-22

**Authors:** Lei Zhao, Cunman Liang, Yan Huang, Guodong Zhou, Yiqun Xiao, Nan Ji, Yuan-Ting Zhang, Ni Zhao

**Affiliations:** 1 Department of Electronic Engineering, The Chinese University of Hong Kong, Hong Kong, China; 2 Department of Biomedical Engineering, City University of Hong Kong, Hong Kong, China; 3 Hong Kong Center for Cerebro-Cardiovascular Health Engineering (COCHE), Hong Kong, China

**Keywords:** Hypertension, Electrical and electronic engineering, Biomedical engineering

## Abstract

Cardiovascular diseases (CVDs) are a leading cause of death worldwide. For early diagnosis, intervention and management of CVDs, it is highly desirable to frequently monitor blood pressure (BP), a vital sign closely related to CVDs, during people’s daily life, including sleep time. Towards this end, wearable and cuffless BP extraction methods have been extensively researched in recent years as part of the mobile healthcare initiative. This review focuses on the enabling technologies for wearable and cuffless BP monitoring platforms, covering both the emerging flexible sensor designs and BP extraction algorithms. Based on the signal type, the sensing devices are classified into electrical, optical, and mechanical sensors, and the state-of-the-art material choices, fabrication methods, and performances of each type of sensor are briefly reviewed. In the model part of the review, contemporary algorithmic BP estimation methods for beat-to-beat BP measurements and continuous BP waveform extraction are introduced. Mainstream approaches, such as pulse transit time-based analytical models and machine learning methods, are compared in terms of their input modalities, features, implementation algorithms, and performances. The review sheds light on the interdisciplinary research opportunities to combine the latest innovations in the sensor and signal processing research fields to achieve a new generation of cuffless BP measurement devices with improved wearability, reliability, and accuracy.

## Introduction

Cardiovascular diseases (CVDs) are the leading cause of mortality and disease burden globally^1^. A report from the European Society of Cardiology states that blood pressure (BP) levels and the risk of stroke or myocardial infarction have a continuous linear relationship^2^, suggesting that BP is a key indicator of the risk of CVD. Conventionally, BP is measured with a sphygmomanometer with an inflatable cuff; the intra-arterial pressure is equal to the cuff pressure, which can be read with a stethoscope or oscillometer. However, auscultatory or oscillometric measurements are intrinsically obtrusive and temporally discrete methods of BP monitoring; thus, the physiological status of a patient may not be recorded in a timely manner, and vital signals even may be missed because some risk patterns are invisible at rest and only occur sporadically during circulatory system stress. Due to the demand for long-term BP monitoring and the inconvenience of the currently available cuff-based methods, efforts have been made to develop wearable and cuffless BP monitoring systems. These systems typically comprise sensors integrated in wearable devices, such as a wristband or earphone, to collect physiological signals. The signals are then fed in a physiological or machine learning model for BP calculation.

In order to select suitable types of sensor signals for BP estimation, it is important to first understand the origin of each type of signal and its relation with cardiac activities. A cardiac cycle consists of a diastolic phase and a systolic phase. During the diastolic phase, the heart chambers are in relaxation and gradually filled with blood returning from the veins. During the systolic phase, heart muscle contracts to produce sufficient pressure in the left ventricle to open the aortic valve, eject the blood into the aorta and pumps blood to the other organs of the body^3^. This process repeats periodically, resulting in BP oscillating between diastolic BP (DBP) and systolic BP (SBP). The ejection process causes a pulse wave and recoil; the former spreads pulse information along the ventricular system^4^ and the latter induces detectable body vibration^5^. The opening and closing events of the aortic and other valves and related cardiac activities can produce a succession of noteworthy sounds^6^. In the meantime, blood volume variation within a body segment (such as the chest) causes changes in local electrical conductivity^7,8^. Behind these mechanical behaviors, electrical activities^3^ of the heart serve as the stimuli and metronome, and the related electrical signals are transmitted to peripheral sites. Based on these cardiac and cardiovascular (CV) activities, different sensing technologies are designed to capture the BP-related information, such as detecting pulse waves by photoplethysmogram (PPG) and tonoarteriogram (TAG)^4^, ejection recoil by ballistocardiogram (BCG) and seismocardiogram (SCG)^5^, heart sound by phonocardiogram (PCG)^6^, local blood volume variation by ultrasound, stroke volume^7^ and cardiac output^8^ by impedance plethysmogram (IPG), and electrical activities of the heart by electrocardiogram (ECG). Numerous mathematical models have been developed to analyze these signals, with different types of models providing different forms of BP estimation output, such as pulse transit time (PTT) based models for beat-to-beat BP estimation and learning algorithms for continuous BP wavefunction extraction.

**Fig. 1:**
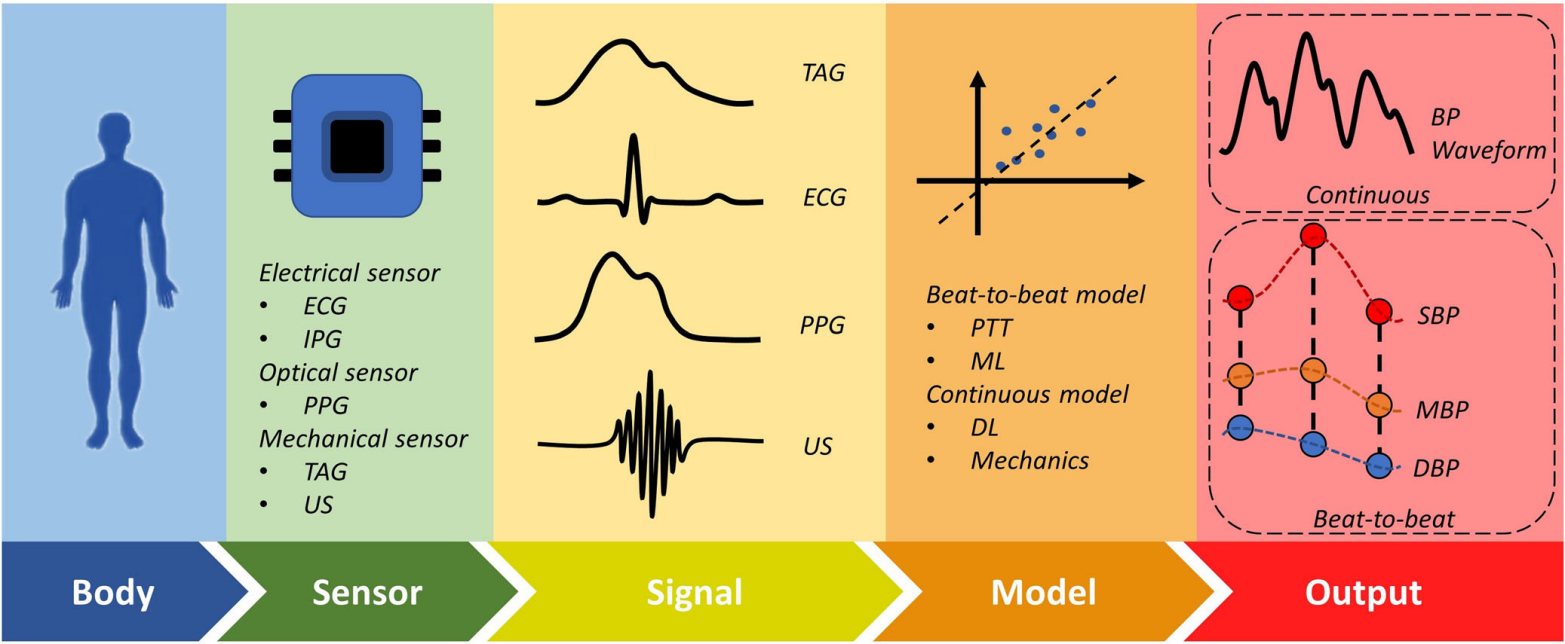
Flow diagram of wearable and cuffless BP monitoring system. Sensors read specific signals from the body and send them to the model for processing and analysis to obtain the BP estimate.

A number of reviews have recently been published to summarize the advances in flexible sensing devices for CV monitoring^4,9^, algorithm development^10–12^, system realization^13–15^, and performance evaluation^16^ for cuffless BP measurements. This review provides a holistic review of these emerging technologies for wearable and cuffless BP monitoring, with the aim of exploring opportunities to merge the merits of device and algorithm development. Figure 1 presents a schematic overview of the key components of a BP measurement event. It can be seen that the measurement mainly relies on a series of critical steps, i.e., signal collection from the sensor end and signal processing and analysis at the back-end calculation units. Accordingly, this review includes flexible sensing technologies for BP monitoring, signals from non-flexible sensors, noise reduction, and estimation models for both the beat-to-beat and waveform BP extraction. Finally, a brief summary of the technology development is provided and the future research directions are discussed.

## Flexible sensing technologies for BP monitoring

### TAG sensors

**Fig. 2:**
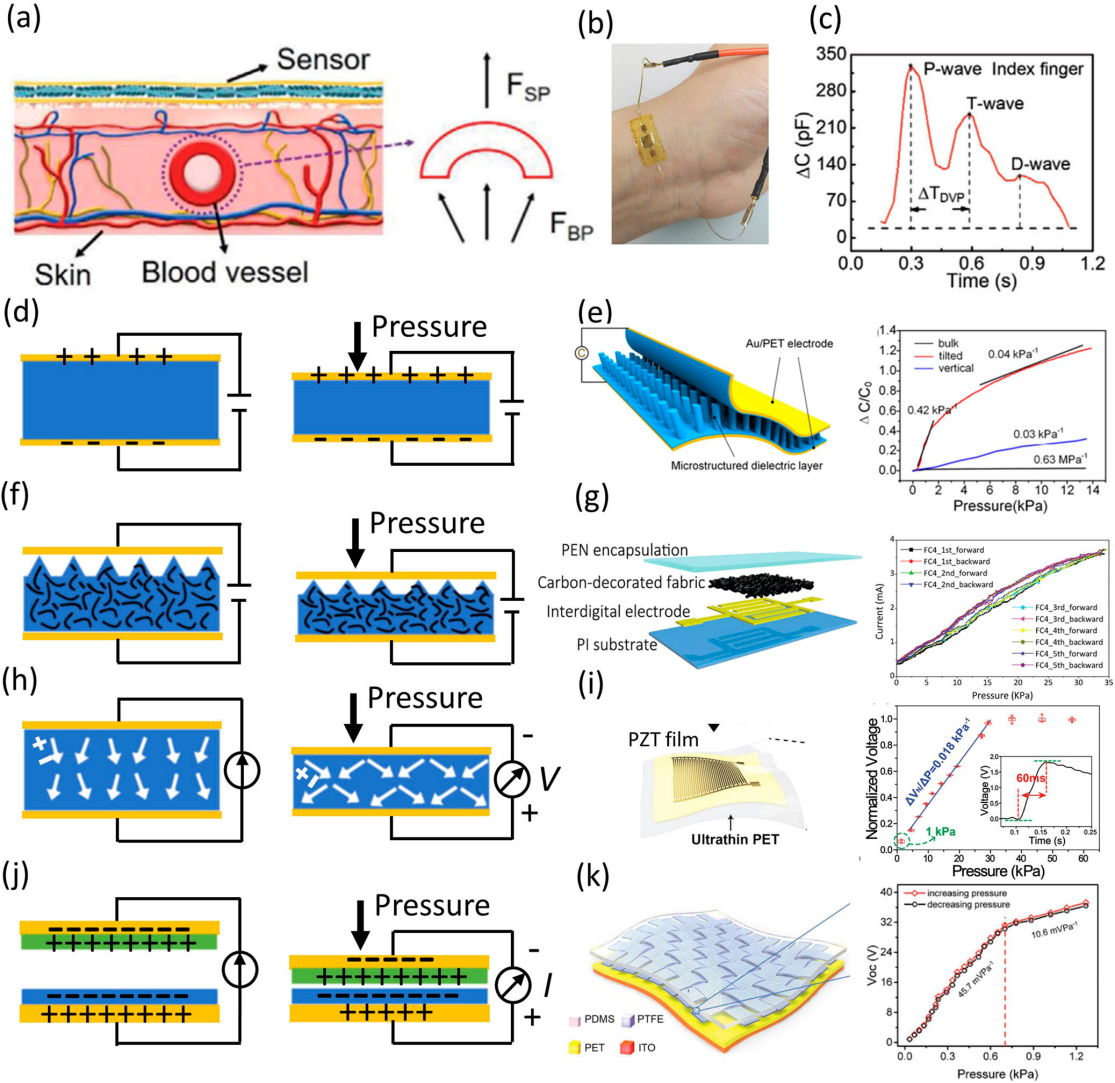
Flexible TAG sensors for epidermal pulse monitoring. **a** Pulse sensing mechanism. Reprinted with permission from ref. ^21^. Copyright 2020 Wiley. **b** A piezoresistive sensor adhering to the human wrist seamlessly. Reprinted with permission from ref. ^22^. Copyright 2017 Wiley. **c** A typical pulse waveform consisting of three characteristic peaks. Reprinted with permission from ref. ^21^. Copyright 2020 Wiley. **d** Piezocapacitive sensing mechanism. **e** A piezocapacitive sensor made of a micropillar dielectric layer, and its pressure transfer curve. Reprinted with permission from ref. ^24^. Copyright 2019 American Chemical Society. **f** Piezoresistive sensing mechanism. **g** A piezoresistive sensor made of a carbon-decorated fabric and its pressure transfer curve. Reprinted with permission from ref. ^25^. Copyright 2015 Wiley. **h** Piezoelectric sensing mechanism. **i** A piezoelectric sensor made of a PZT film and its transfer curve. Reprinted with permission from ref. ^26^. Copyright 2017 Wiley. **j** Triboelectric sensing mechanism. **k** A triboelectric sensor made of woven PTFE stripes and a PET film, together with its sensing mechanism. Reprinted with permission from ref. ^27^. Copyright 2018 Wiley.

TAG is a continuous arterial BP-related signal recorded by a pressure sensor or estimated by other cuffless measuring techniques^17^. Flexible TAG sensors are devices that can convert pressure inputs into electrical outputs. Compared with traditional MEMS-based rigid sensors, the most crucial advantage of flexible TAG sensors is their excellent conformability to curved or soft surfaces, such as human skin^18–20^. Due to this advantage, they can be used as wearable sensors for monitoring key physiological signals, such as epidermal pulses. The BP on the vascular wall of arteries distributed in superficial tissues can be transmitted to the skin surface (Fig. 2a)^21^. A TAG sensor adhering to the skin surface can thus capture the tiny pressure variations caused by the pulse wave (Fig. 2b)^22^. This cuffless method can realize continuous pulse monitoring with little user discomfort. A typical pulse waveform comprises three characteristic peaks: the percussion wave (P-wave), tidal wave (T-wave), and diastolic wave (D-wave), as shown in Fig. 2c^21^. The P-wave is caused by the initial systolic spike due to blood ejection from the contracting left ventricle. The T-wave and D-wave are caused by blood flow reflection from the upper and lower body, respectively^23^.

Four pressure sensing mechanisms are commonly used: piezocapacitive (Fig. 2d), piezoresistive (Fig. 2f), piezoelectric (Fig. 2h), and triboelectric (Fig. 2j) mechanisms. Piezocapacitive and piezoresistive materials indicate pressure exerted on the material through changes in capacitance (Fig. 2e) and resistance (Fig. 2g)^24,25^, respectively. Piezoelectric and triboelectric methods transduce pressure into a change in electric potential (Fig. 2i, k) and are thus self-powered devices^26,27^. All TAG sensors have three essential components: sensing materials, flexible substrates, and electrodes. Flexible substrates are typically made using thin films of polyimide (PI)^28,29^, polydimethylsiloxane (PDMS)^30^, and polyethylene terephthalate (PET)^31^. Flexible electrodes are usually thin films made of conductive metals^32^ and metal oxides (e.g., indium tin oxide (ITO))^33^. Sensing materials vary between sensor types and have been discussed in depth in several review articles^34–36^. The general advantages and disadvantages of sensor types are summarized in Table 1. The sensitivity of triboelectric and piezoelectric sensors differs greatly from other sensors; thus, they can only be compared with each other.

**Table 1:** Comparison of different types of TAG sensors.

Types	Advantages	Disadvantages
Piezocapacitive (without electric double layer)	Suitable for both dynamic/static measurement, environmental stability, long linear range available	Low sensitivity
Piezocapacitive (with electric double layer)	Suitable for both dynamic/static measurement, environmental stability, high sensitivity available	Trade-off between linear range and sensitivity
Piezoresistive	Suitable for both dynamic/static measurement, environmental stability, high sensitivity available	Trade-off between linear range and sensitivity
Piezoelectric	Selfpowered, short linear range	Unsuitable for static measurement, low sensitivity (compared with triboelectric type)
Triboelectric	Selfpowered, short linear range, high sensitivity (compared with piezoelectric type)	Unsuitable for static measurement, sensitive to humidity

### ECG and bioimpedance electrodes

**Fig. 3:**
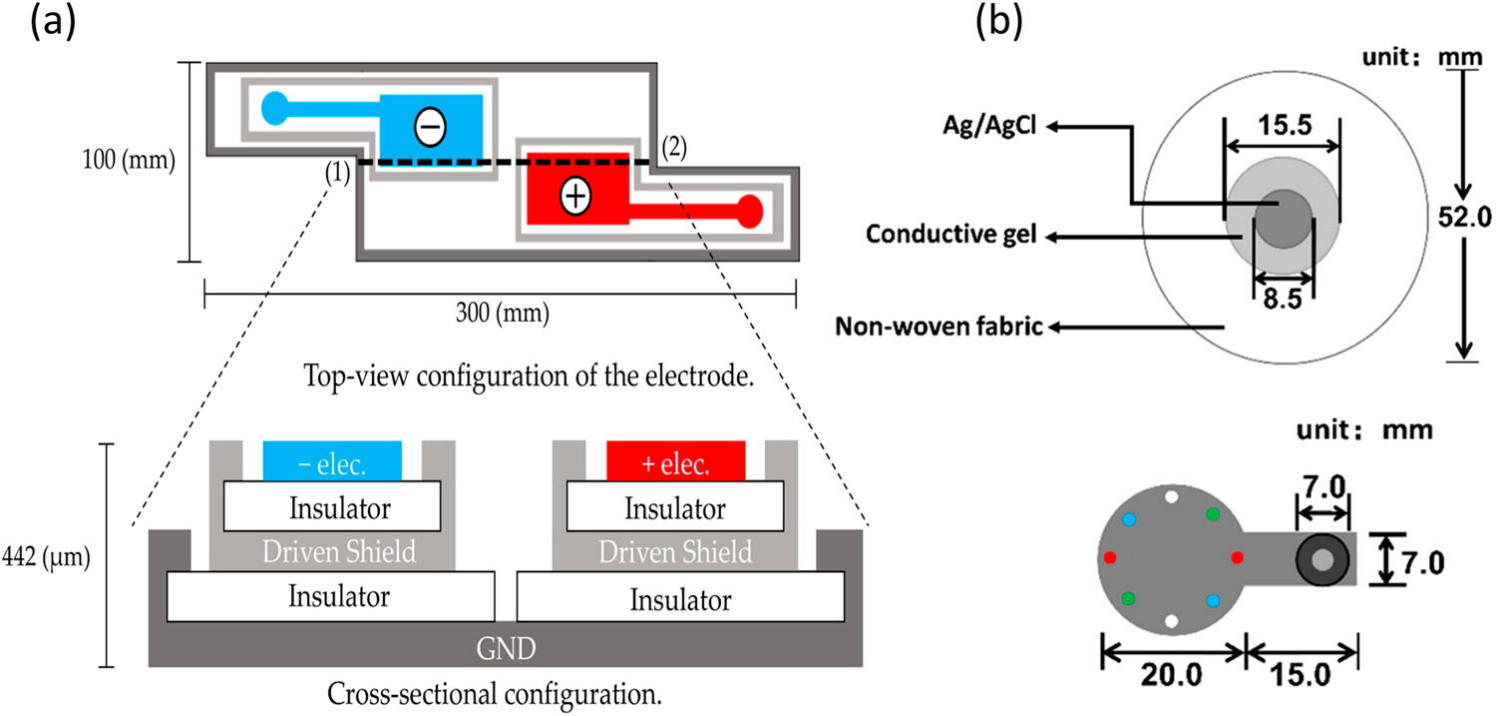
ECG electrodes. **a** Schematic diagram of in-pillow capacitive cloth electrodes^40^. **b** Schematic diagrams of the standard Ag/AgCl electrode and prepared electrode^78^.

An ECG signal is a record of the potential difference between conductive electrodes across the heart. These electrodes (Fig. 3) are attached to the skin of the arms, legs, and chest. Three types of ECG electrodes are used, namely wet^37–39^, dry, and capacitively coupled electrodes^40^. Wet and dry electrodes are directly connected to the skin and can measure skin impedance via conductive material. By contrast, capacitively coupled electrodes are attached to the skin with an insulating layer, and they measure the capacitively coupled charge with local electronics. Wet electrodes, such as the clinically used Ag/AgCl electrode, can provide a high-quality signal; however, they require electrolytes that may cause skin irritation. This electrolyte also decays over time, requiring replacement. Thus, wet electrodes are unsuitable for long-time ECG monitoring. By contrast, dry electrodes can be used for long-term ECG monitoring; however, the signal quality is comparatively poor due to the high impedance of the skin. Capacitively coupled electrodes with integrated electronics can provide high-quality measurements; however, the cost of fabricating these electrodes is high. The ability to maintain morphological waveforms under both dry and water-immersed conditions is essential for daily ECG monitoring^41^. In 2018, Ramasamy and Balan^42^ summarized the performances and fabrication methods for these three types of ECG electrodes. In 2020, Gandhi and Raghava^43^ reviewed the fabrication methods in a targeted manner. The materials for ECG electrodes should be conductive, biocompatible, and time-durable. Details regarding the conductive materials are listed in Table 2.

**Table 2:** Conductive materials used in ECG electrode studies.

Materials	Substrate	Type	Reference
Laser-induced graphene	PDMS	Dry	^150^
Conductive fabric	Polyester	Capacitive	^40^
Carbon pencil lead	Tape	Dry	^151^
Copper tapes	Paper	Dry	^152^
Tylson conductive silver fabrics	Paper	Dry	^152^
PVA/GNR	Ag/AgCl	Wet	^37^
Al-doped TiO_2_	Ag	Dry	^153^
CVD graphene	EVA/PET plastic film	Dry	^154^
Silver	Cowhide	Dry	^78^
PEDOT/PSS/PVDF/Nanofiber Carbon	Glass	Dry	^155^
PPy-Leather	Leather	Dry	^156^
PEDOT: PSS Hydrogel	Wound dressing, gauze bandage, sanitary napkins sponge, filter paper	Wet	^39^
MWCNT	PDMS	Dry	^157^
Conductive hydrogel	Conductive hydrogel	Wet	^38^
Carbon black conductive paste	TPU films	Dry	^158^
Au	Stainless steel substrate	Dry	^79^

Electrodes for ECG can also be used for bioimpedance monitoring, such as IPG monitoring^44^. IPG, also known as bioimpedance plethysmography (BPG)^45^, can provide useful features for BP calculation algorithms^46,47^. For instance, impedance cardiogram (ICG), a special IPG waveform that measures the thoracic electrical bioimpedance, is often used to calculate the pre-ejection period. In bioimpedance monitoring, two pairs of electrodes are required: one for AC current injection (some methods use a short current pulse^47^) and the other for voltage difference sensing^44^. In IPG, the electrode placement is flexible; that is, the electrode pairs need not cross the heart as ECG electrodes do. For example, IPG electrodes can be placed on the neck^44^, wrist^48,49^, arm^7^, leg^50^, feet^51^, or on multiple body sites^45^.

### Optical sensors

**Fig. 4:**
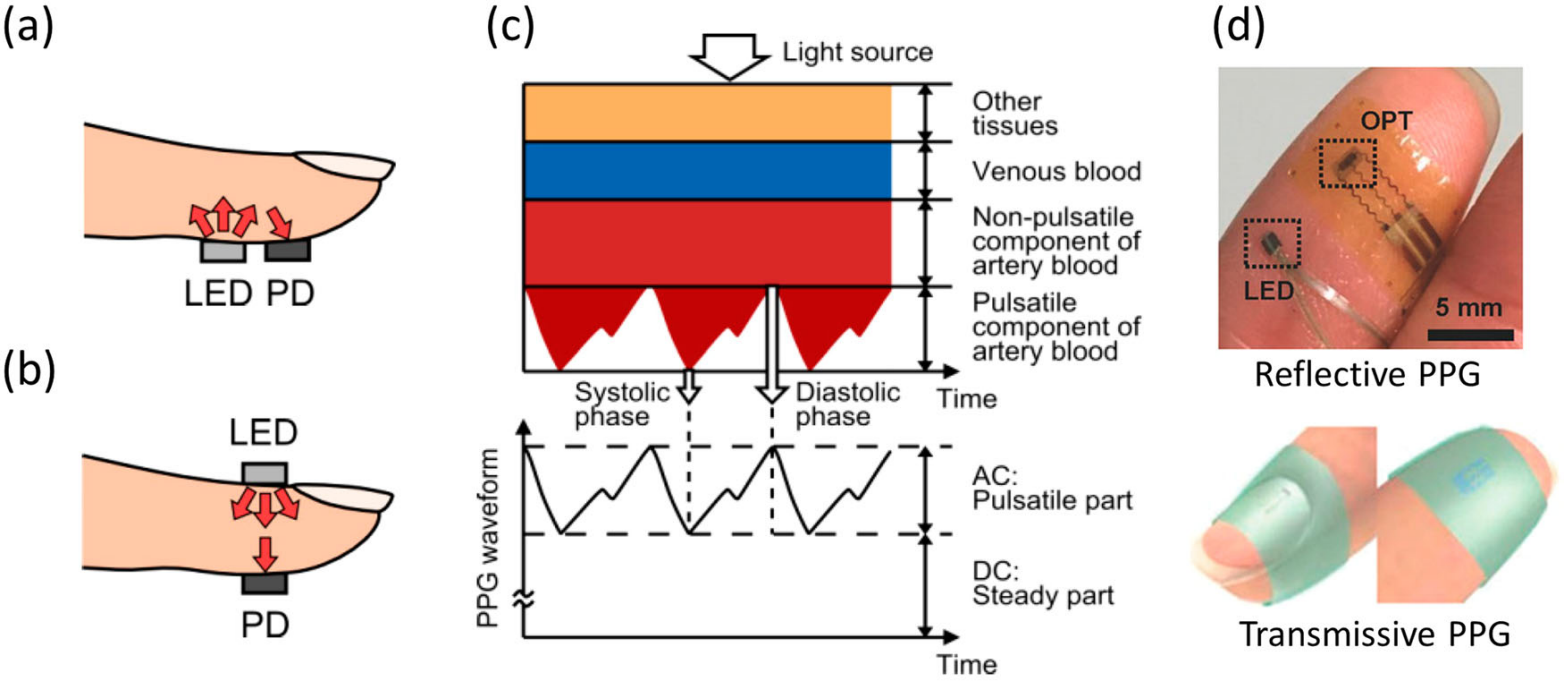
Working principle of PPG^195^. **a** Reflective mode. **b** transmitting mode. **c** example of PPG signal. **d** Reflective mode epidermal and transmissive mode flexible hybrid organic/inorganic NIR PPG for BP monitoring. Reprinted with permission from ref. ^59^. Copyright 2017 Wiley.

PPG devices comprise light sources and photodetectors. The light sources are typically light-emitting diodes (LEDs), and the photodetectors convert light into PPG signals (Fig. 4a, b). A PPG signal from a photodetector indicates the amount of optical absorption or reflection, which is responsive to the change of blood volume in the optical path. In practice, PPG sensors are routinely placed at peripheral body sites, such as the finger, earlobe, or forehead, to monitor cardiac-induced changes in microvascular blood volume^52^. Light of ~510–590 nm in wavelength is the primary contributor to the pulsatile component of the reflected light, and infrared light has the greatest penetration depth. Thus, green (565 nm) or yellow (590 nm) light is typically used in reflective PPG sensors, whereas red (680 nm) or near-infrared (810 nm) light is used in transmissive PPG devices. Multiwavelength PPG (MWPPG) can also be used to calculate BP with PTT^53^.

Wearable PPG devices, such as eyeglasses^54^ and bulky pulse oximeters^55^, have been commercialized for CV monitoring. Scientists have also developed state-of-the-art conformal and unobtrusive PPG sensors with new materials and designs. In 2018, Lee et al.^56^ designed a ring-like organic LED (OLED) and organic photodiode (OPD) patch that maximized PPG signals in the reflective mode for use as a pulse oximeter. Vital sign tracking can even be achieved under ambient light without LED sources^57,58^. In 2019, Polat et al.^57^ successfully demonstrated flexible and transparent wearables based on graphene sensitized with semiconducting quantum dots in a phototransistor (PT) structure. They achieved heart rate and arterial blood oxygen saturation monitoring under ambient light. In 2020, Han et al.^58^ also produced a pulse oximeter by adding passive optical filters to OPDs. However, estimating BP by applying only PPG techniques is not yet mature; integrating other physiological signals enhances the accuracy of these methods. In 2017, Xu et al.^59^ combined ECG signals with a flexible organic-inorganic hybrid near-infrared PPG sensor integrating a low-power, high-sensitivity organic PT and a high-efficiency inorganic LED for real-time heart rate variability and BP tracking in both the reflective and transmissive modes (Fig. 4d). Future device optimizations enabling high-precision monitoring of pulse waves, along with improved algorithms, could spur the adoption of PPG for CV monitoring, including for BP estimation^60^.

### Ultrasound sensors

Ultrasound sensors for wall tracking are a promising, noninvasive device for BP waveform monitoring and have increasingly attracted the attention of researchers^61^. Compared with other noninvasive methods, such as PPG and TAG, the ultrasound wall-tracking technique has a higher penetrating capability, which can be used to track the BP waveform of vasculature embedded in deep tissues^62^. However, current commercial ultrasound probes are heavy, rigid, and bulky; moreover, the ultrasound probe must be held and stabilized by an experienced operator to achieve a reliable acoustic coupling interface^63,64^. These problems inevitably lead to inaccurate results because the probe compresses local vasculatures and changes their distension behavior. Therefore, conventional ultrasound probes are unsuitable for the long-term monitoring of BP waveforms.

**Fig. 5:**
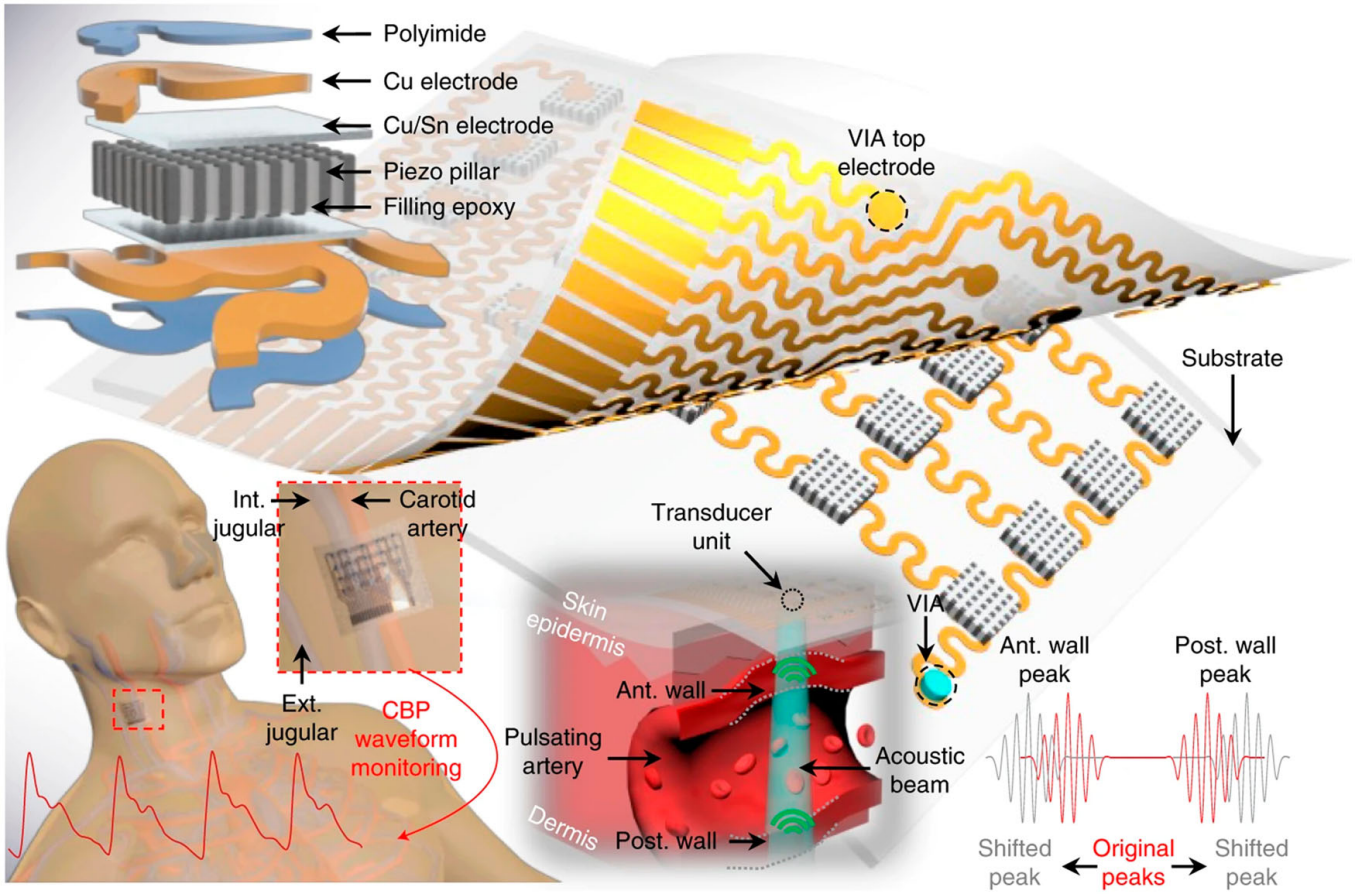
Schematic diagram of the stretchable ultrasound device. Reprinted with permission from ref. ^70^. Copyright 2018 Nature.

Flexible wearable devices with mechanical properties similar to those of skin enable long-term, continuous, and timely monitoring of vital signs^65–67^. Ultrasound sensors that are flexible and wearable are attractive solutions to the aforementioned problems in ultrasound-based methods. In 2022, Wang et al.^68^ reported a bioadhesive ultrasound (BAUS) device that utilizes a couplant consisting of a soft yet tough hydrogel covered by a thin antidehydrating elastomer to achieve strong adhesion between the ultrasound probe and skin, thus allowing for long-term continuous imaging and BP tracking. In 2021, Sempionatto et al.^69^ proposed a noninvasive skin-worn ultrasound sensor for monitoring BP and heart rate. This ultrasound sensor contained 1 × 8 ultrasonic transducers with a kerf of 0.2 mm; each transducer had a size of 4 mm × 1 mm × 0.25 mm. Styrene-ethylene-butylene-styrene (SEBS) block copolymer was used as the substrate, superstrate, and filler. Stretchable electrodes were produced with silver ink and could be integrated in the SEBS substrate. Each ultrasonic transducer vibrated in thickness mode at a fixed frequency of 7 MHz. The sensor should be located over the targeted vessel to measure the BP waveform. To easily place the sensor at the correct position, in 2018, a flexible ultrasound sensor with a 4 × 5 transducer array (*f* = 7.5 MHz) was developed by Wang et al.^70^ (Fig. 5). An “island–bridge” layout with a serpentine electrode, produced by stacked polyimide and Cu in a bilayer fashion, was exploited to produce a stretchable device. The transducers could individually generate and receive ultrasound waves. The array design enables sensing and monitoring by a transducer located over the targeted vessel without manual positioning. To protect the device from possible corrosion by sweat, the transducers were encapsulated with silicone; this encapsulation can also be used to produce a conformal device that makes excellent contact with the skin surface.

Little research has been performed on flexible and wearable ultrasound sensors for monitoring BP waveforms. Integrating the back-end functions of the devices, such as electronic control, signal processing, and power sources, in a stretchable and wearable ultrasound sensor remains challenging. Thus, their practical applications are limited, especially for the long-term and continuous monitoring of BP waveforms. Moreover, although ultrasound sensor arrays with small transducers and small pitches have high measurement accuracy, these sensors are difficult to fabricate with existing technologies^71^.

## Other signals from non-flexible sensors

Apart from the biosignals that have already been measured with flexible sensors, there are other cardiac activity-induced signals that can readily be captured by portable, wearable, or furniture-integrated sensors and may be used to enhance or expand the BP monitoring technologies. For instance, BCG/SCG can indicate CVD-induced slow and longitudinal changes in cardiac functions^5^ and therefore used to trigger recalibration of BP models. It has been demonstrated that BCG/SCG signals can be obtained by weighing scales, bed-based^72^, and chair-based devices, as well as accelerometer-embedded wearable devices^5^. PCG records heart sounds produced by cardiac activities, such as the opening and closing movements of the mitral, tricuspid, aortic, and pulmonary valves, and some key points, such as S1 and S2, can be used for BP estimation^6,73^.

## Noise reduction techniques

Power-line interference (PLI), external electromagnetic (EM) interference, baseline wander (BW), and motion artifacts (MAs) are the most common sources of noise in biosignals^5,8,13,74–76^. PLI superposes 50/60 Hz sinusoidal noise and its higher harmonics associated with the frequency of power lines^77^ on almost all types of signals. EM noise often appears in ECG and PPG signals but can be removed or reduced through shielding. BW refers to the shift of signal baseline due, for example, to the environment or intrinsic property change of the sensor unit, and normally occurs in a low-frequency band. MAs are signal noise induced by respiration, muscle movements, poor skin-sensor/electrode contact, etc.^45,75,78^. Noise reduction is an indispensable component for wearable cuffless BP monitoring systems. The most broadly used denoising tool is filter, which has been typically applied to suppress the noise of a certain frequency range in TAG^75^, ECG^40,78,79^, IPG^7,44,49^, PPG^13,75^, ultrasound^76,80^, and BCG^72,81^ signals. While filtering can serve as a universal tool for almost all types of sensing modalities, there have also been signal-specific denoise methods developed to improve the noise removal efficiency and accuracy. Satija et al.^74^ proposed an automated ECG noise detection and classification system for unsupervised ECG analysis, during which a decision rule-based algorithm is executed to detect the presence of noises and classify input ECG signals into six groups, namely noise-free ECG, ECG + BW, ECG + MA, ECG + PLI, ECG + BW + PLI, and ECG + BW + MA for more accurate denoise treatment. The method achieved average sensitivity, positive predictivity, and classification accuracy of 98.93%, 98.39%, and 97.38% on a large collection of ECG signals taken from five standard databases. Poh et al.^82^ designed a PPG sensing system with adaptive noise cancellation (ANC) which employed an embedded accelerometer as the reference signal sensor to remove MAs and achieved significantly improved heart rate tracking performances. Ibrahim and Jafari^83^ developed a generalized extreme studentized deviate (GESD) algorithm that exploits the inter-channel variance of four-channel IPG signals extracted in small areas to remove MAs. Pashaei et al.^80^ proposed a pre-amplifier design in a flexible ultrasound probe circuit to maintain the signal-to-noise ratio (SNR) during signal transmission from the probe to the receiver, thus enhancing the detection sensitivity to small organs, e.g., blood vessels. ~5 dB reduction in noise figure was visible around the nominal imaging frequency of 5 MHz (transducer resonance) when comparing simulation performance of the proposed active probe with the passive probe. There have also been several noise reduction strategies for BCG/SCG measurements, which are summarized in a recent review by Inan et al.^5^.

## Beat-to-beat BP estimation models

**Fig. 6:**
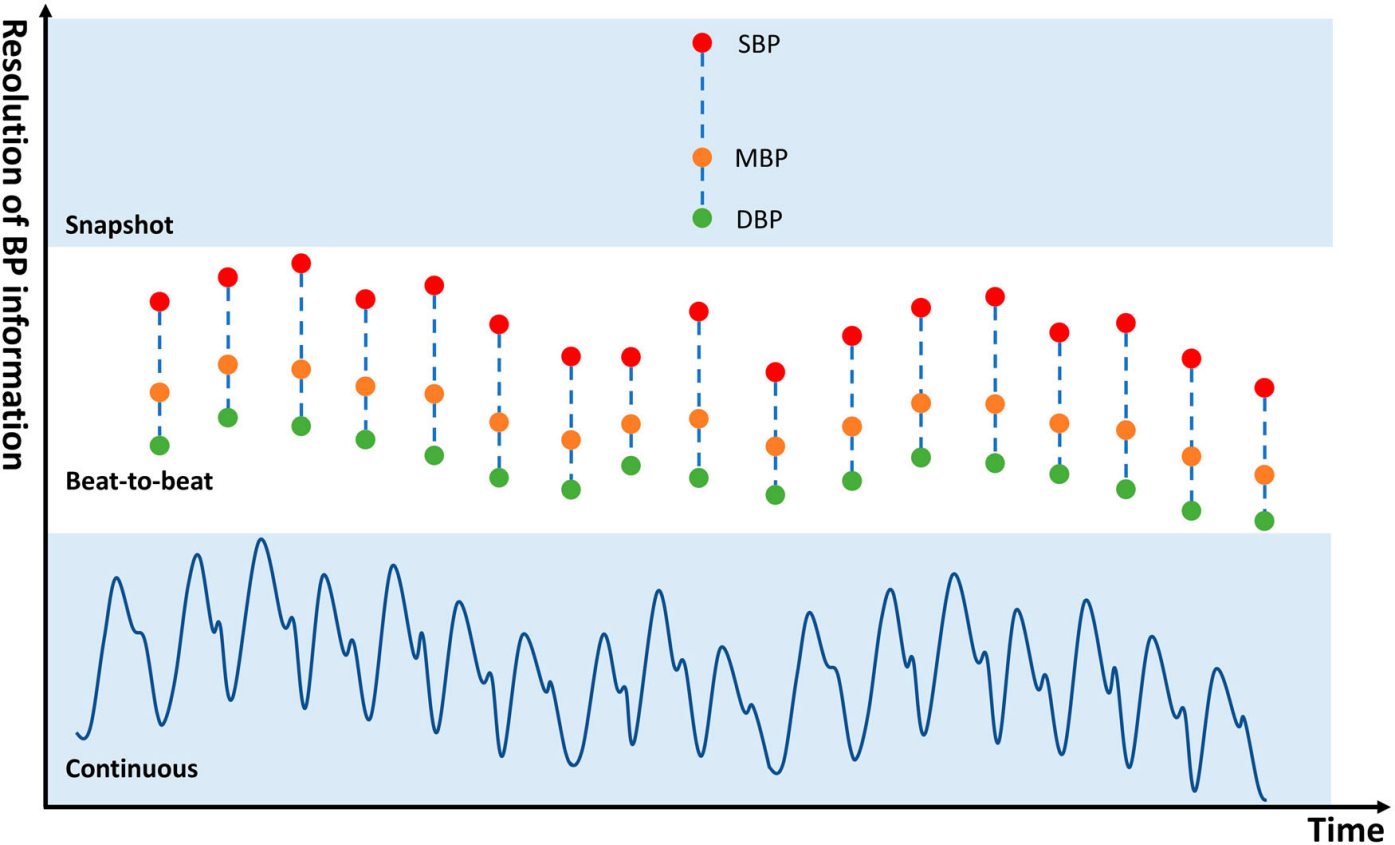
Schematic diagram of BP information in different temporal resolutions. From fine to coarse-grained: continuous BP waveform, beat-to-beat BP, and snapshot BP.

BP estimation techniques are divided into three categories in accordance with the temporal resolution of the output BP information: snapshot, beat-to-beat, and waveform continuous techniques (Fig. 6). Conventional cuff-based methods, such as auscultatory or oscillometric measurement, are used to measure snapshot BP. Thus, beat-to-beat and waveform continuous methods, as in wearable and cuffless devices, are reviewed in this section. Beat-to-beat models provide an estimation of BP parameters at each heartbeat, whereas the continuous models output continuous BP waveforms.

### PTT-based methods

**Fig. 7:**
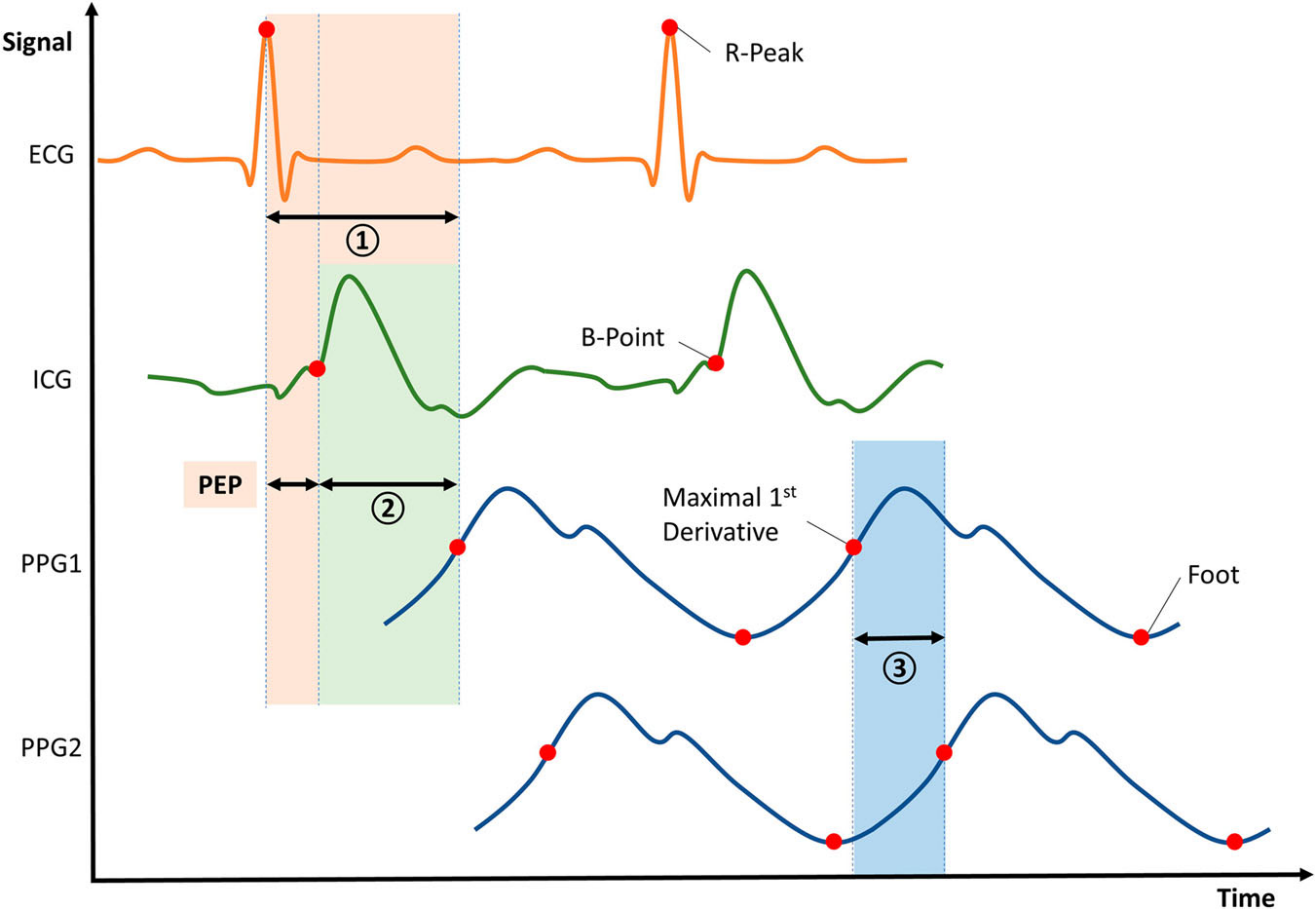
Illustration of two mainstream definitions of PTT and PAT. Mukkamala et al.^84^: ①—PAT, ②③—PTT; Ding et al.^85^: ①—PTT_EP_, ②—PTT_IP_, ③—PTT_PP_, or PAT.

The PTT-based analytical model is a dominant beat-to-beat BP estimation method (Table 3). There are two mainstream definitions of PTT and pulse arrival time (PAT), as illustrated in Fig. 7. Mukkamala et al.^84^ suggest that PTT is the time period for the pressure wave to travel between two arterial sites and PAT is the time delay between the ECG waveform and its adjacent distal arterial waveform. Meanwhile, Ding et al.^85^, on the basis of the original meaning of “transit” and the history of relevant research, suggest that PTT should be the time it takes the pulse signal to carry the pulse wave information from one location to another in a CV system, while that PAT refers to the pulse in the same form of energy from the starting point to the arrival point; accordingly, PTT is marked as ①, ②, and ③, while ③ also refers to PAT, as shown in Fig. 7. Moreover, signals used to calculate PTT can be labeled in the subscript to avoid definition ambiguity. With a proper definition, the pre-ejection period (PEP) can be either included in or excluded from PTT^85^. The Moens–Korteweg equation^86^ ${PWV}=\sqrt{\frac{E{h}_{0}}{2\rho {R}_{0}}}$ and Hughes equation^87^ $E={E}_{0}\exp \left(\xi P\right)$ are fundamental to PTT analysis; ${PWV}$, $E$, ${h}_{0}$, $\rho$, and ${R}_{0}$ are pulse wave velocity, the elastic modulus at pressure $P$, artery thickness, blood density, and artery radius; ${E}_{0}$ and $\xi$ are the elastic modulus at zero pressure and the material coefficient of the artery. The underlying physiological model assumes that the artery stiffens as the BP increases (Hughes equation), leading to an increase in PWV or a decrease in PTT (M-K equation). In the following, we summarize the typical signal combinations used for BP model implementation.

**Table 3:** Conclusion of PTT-based analytical BP estimation methods.

Modalities	Ref.	Model		#Sub.	Performance
ECG + PPG	^90^	${DBP}=\frac{{SB}{P}_{0}}{3}+\frac{2\,{{DB}P}_{0}}{3}+A\cdot \mathrm{ln}\left(\frac{{PT}{T}_{{W}_{0}}}{{PT}{T}_{W}}\right)-\frac{\left({SB}{P}_{0}-{DB}{P}_{0}\right)}{3}\frac{{PT}{T}_{{W}_{0}}^{2}}{{PT}{T}_{W}^{2}}$ ${SBP}={DBP}+\left({SB}{P}_{0}-{DB}{P}_{0}\right)\frac{{PT}{T}_{{W}_{0}}^{2}}{{PT}{T}_{W}^{2}}$	(1)	85	Difference = $0.6\pm 9.8$ mmHg and $0.9\pm 5.6$ mmHg for SBP and DBP
	^92^	${PWV}=\frac{{BDC}\times h}{{PTT}}$ ${SBP}={P}_{1}\times {PWV}\times {e}^{{P}_{3}\times {PWV}}+{P}_{2}\times {PW}{V}^{{P}_{4}}-\left({SB}{P}_{{cal}}-{SB}{P}_{0}\right)$	(2)	50	Individual correlation coefficient r ranges from $0.69$ to $0.99$ in the validation period. The repeated measurements result in ${\rm{r}}=0.83.$
	^93^	${DBP}={DB}{P}_{0}\cdot \frac{{PI}{R}_{0}}{{PIR}}$ ${SBP}={DB}{P}_{0}\cdot \frac{{PI}{R}_{0}}{{PIR}}+P{P}_{0}\cdot {\left(\frac{{PT}{T}_{0}}{{PTT}}\right)}^{2}$	(3)	27	Difference = $-0.37\pm 5.21$, $-0.08\pm 4.06$, and $-0.18\pm 4.13$ mmHg for SBP, DBP and MBP; MAD = $4.09$, $3.18$, and $3.18$ mmHg
MWPPG	^94^	${MBP}={HR}\cdot \left({k}_{1}\cdot {DRPPG\; TD}+{b}_{1}\right)$ ${PP}={MBP}\cdot \left({k}_{2}\cdot \frac{{t}_{\tau }}{{HP}}+{b}_{2}\right)$	(4)	20 + 4	20 subjects are for Finometer test, and MADs for SBP and DBP are $2.85$ and $1.75$ mmHg. 4 subjects are for the arterial line test, and MAD is $2.1$ mmHg.
IPG + PPG	^96^	${DBP}={DB}{P}_{0}+\rho \frac{{D}^{2}}{{PT}{T}^{2}}{ln}\left[1+K\left({Z}_{max\,0}-{Z}_{min }\right)\right]$ ${SBP}={SB}{P}_{0}+\rho \frac{{D}^{2}}{{PT}{T}^{2}}{ln}\left[1+K\left({Z}_{max \,0}-{Z}_{max }\right)\right]$ $K=\frac{exp \left(\frac{{SB}{P}_{0}-{DB}{P}_{0}}{\rho }\frac{{PT}{T}_{0}^{2}}{{D}^{2}}\right)-1}{{Z}_{max \,0}-{Z}_{min \,0}}$	(5)	15	The BP reference was measured by Oscar 2 system (SunTech Medical, USA). The average coefficient is $0.88\,\pm \,0.07$ mmHg for SBP and $0.88\,\pm \,0.06$ mmHg for diastolic BP (DBP). RMSE is $8.47\,\pm \,0.91$ mmHg and $5.02\,\pm \,0.73$ mmHg for SBP and DBP, respectively.
TAG + PPG	^75^	${SBP}=\frac{2}{B}{ln}\frac{A}{{{PTT}}_{s}}$ ${DBP}=\frac{2}{B}{ln}\frac{A}{{{PTT}}_{D}}$	(6)	15	MAD = $2.62$ and $1.36$ mmHg for SBP and DBP

(1) ECG + PPG: Dating back to 1978, Obrist et al.^88^ evaluated 114 human subjects and reported that the PTT varied consistently with SBP but not DBP. In the 2000s, Chen et al.^89^ demonstrated that changes in PTT are linearly related to changes in SBP over a short period. Based on this high-frequency variation, a long-term estimation can be achieved by including a low-frequency baseline extracted from intermittent BP measurements. Poon and Zhang^90^ developed a technique for simultaneously estimating DBP and SBP. These results suggest that cuffless methods are promising for wearable healthcare. A 6-month study conducted by Wong et al.^91^ suggested that PTT-based techniques require an auxiliary module or an independent subalgorithm for intermittently tracking baseline changes. In the 2010s, Gesche et al.^92^ constructed a nonlinear model to describe the relation between PWV and BP and proposed a one-point calibration scheme for the model. The results indicated that the PWV-based estimation method has the potential for BP monitoring; however, customized individual corrections are required due to subject differences. Ding et al.^93^ proposed an indicator called photoplethysmography intensity ratio (PIR), which takes vasomotor tone into account. The inclusion of PIR in PTT-based BP estimation improved its accuracy^93^.

(2) MWPPG: On the basis of conventional PPG signals and PTT-based BP estimation methods, Liu et al.^53^ developed an MWPPG sensor and analyzed its mechanism in 2016. By using the signals extracted with these MWPPG sensors, Liu et al.^94^ proposed a depth-resolved approach for continuous BP monitoring in 2018.

(3) IPG-based PTT: In 2018, Huynh et al.^95^ proposed and examined a miniaturized bioimpedance device consisting of two sets of four-electrode interfaces attached around the wrist of a subject to measure IPG-based PTT in a 0.5 × 1.75 cm^2^ area. Leveraging both the PPG and IPG signals, they included changes in the cross-sectional area of blood vessels in a PTT-based model and improved the BP estimation accuracy^96^. It is worth noting that BP can also be estimated using IPG-ECG^51^, IPG-PPG^49,97^, IPG-IPG^8^ (including IPG + ICG^45^) combinations, or even single-channel IPG^48^. Several studies have evaluated the performance of PTT-based BP estimates using IPG + ECG and PPG + ECG and reported that the former is superior to the latter in a manner^7,44^.

(4) BCG-based PTT: In 2015, He et al.^98^ employed a wearable device to monitor BCG, ECG, and PPG simultaneously and avoid interference from PEP by using BCG and PPG to determine PTT. Kim et al.^81^ showed that BCG can serve as a proximal timing reference for PTT calculation, which may outperform the ECG-based PAT for BP estimation.

(5) TAG + PPG: In 2022, Samartkit et al.^75^ integrated PPG and TAG to monitor BP and heart rate. The TAG signal was extracted by a lead zirconate titanate (PZT) piezoelectric sensor. In contrast to general PTT calculation, where only the time interval between two adjacent systolic peaks is used, their modified PTT technique measures PTTs for SBP and DBP separately.

It is worth noting that a recent report from Microsoft Research compared different cuffless BP extraction models over more than 1000 subjects and found that the commonly used PAT and pulse wave (PWA) analysis methods do not offer meaningfully more accurate estimation results than those obtained from baseline models^99^. This finding may reflect the fundamental limitation of the PTT/PWA methods in relying on overly simplified blood vessel-regulated models and calls for developing more comprehensive cuffless BP measurement models with strong theoretical underpinnings^100^.

### Machine learning-based methods

**Fig. 8:**
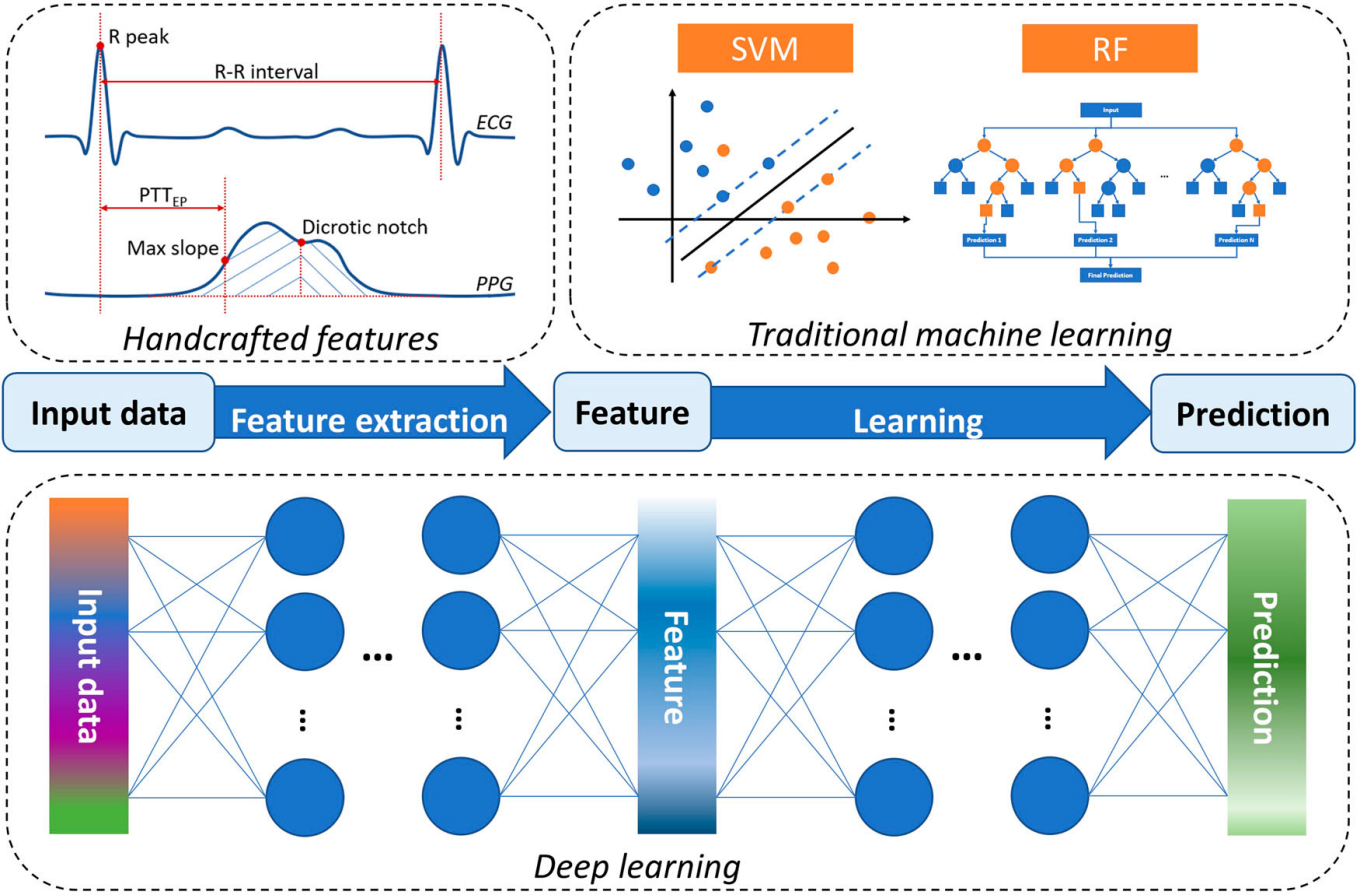
General pipeline of machine learning algorithms for BP monitoring. Traditional ML contains a separate feature extraction step, while DL can perform end-to-end inference.

Machine learning (ML) is a powerful tool for algorithmic beat-to-beat BP estimation. ML-based BP estimation methods are categorized into traditional and deep learning (DL) algorithms (Fig. 8). In contrast to traditional ML algorithms, DL methods can perform end-to-end predictions and have a layered model structure with greater depth. The common ML methods, such as linear regression (LR), support vector machine (SVM), k-nearest neighbors (KNN), decision tree (DT), and random forest (RF), are discussed in this review. DL methods include convolutional neural networks (CNN), recurrent neural networks (RNN), and their hybrids.

#### Traditional ML methods

In contrast with numerous hidden layers and the strong fitting ability of DL, traditional ML models have better interpretability and generalization. Typically, the first step in training a traditional ML model is handcrafted feature extraction for later analysis. Some commonly used features for BP estimation are listed in Table 4.

**Table 4:** Summary of handcrafted feature extraction methods for potential BP monitoring.

Categories	Descriptions	Modalities	Ref.
Waveform features	The following features are extracted from the waveform of a signal or its 1st/2nd derivative: • Peak/valley/key point amplitude • Amplitude difference (height) between two key points • Time duration (width) between two key points • The area under the waveform during a certain period • Angle/slope	PPG	^111,159–176^
		ECG	^173–175,177,178^
		US	^179–181^
		TAG	^182,183^
		IPG	^8^
PTT/PAT/PWV-based features	The time interval between two reference points in two signals of the same or different types. PWV-based features take both transit time and distance into consideration. Reference points include but are not limited to: • ECG R-peak • PPG peak/foot/maximum 1st derivative point • IPG B-point • TAG systolic/diastolic peaks, etc.	TAG + TAG	^27^
		PPG + ECG	^105,164,173,175,177,184–187^
		PPG + TAG	^188^
		ECG + TAG	^17,182^
		ECG + IPG	^7^
		IPG + IPG	^8^
		PPG + IPG	^49,97^
Frequency domain features	Signals are transformed to the frequency domain to extract spectral features.	PPG	^170^
PIR-based features	The ratio between signal systolic and diastolic amplitude is an indicator for BP.	PPG	^175,177,189–191^
		TAG	^182^
PAR	PPG acceleration ratio	PPG	^192^
Statistical features	Statistics of raw signal sequences or extracted parameters, e.g., the standard deviation of RR intervals	PPG	^193^
		ECG	^194^

LR is the most frequently used traditional ML algorithm in BP estimation. Some common optimization methods for LR include least-square and maximum-likelihood estimation. In 2022, Natarajan et al.^101^ integrated PTT features with PPG data extracted from the finger, ear, and toe to train LR models and eliminated errors in BP prediction. In 2022, Figini et al.^102^ used PTT and heart rate as features to train several regression algorithms, including LR, for predicting SBP and DBP. In 2021, Mamun^103^ applied a penalty-based regression outperforming the conventional LR in both SBP and DBP prediction in terms of the mean absolute error (MAE). In 2021, Liu et al.^104^ evaluated multiple LR models with data from a maximal exercise stress test to achieve a robust cuffless prediction of BP during exercise.

SVM is a powerful and widely used ML method. In SVM, a model is optimized by maximizing the margin between different subspaces in the feature space to achieve accurate classification. SVM models can be used to predict BP with individual-level data^104,105^ or public datasets^106,107^. In 2017, Zhang et al.^108^ applied SVM to classify clean and noise-polluted ECG cycles. Plug-and-play SVM modules are easily available and can accelerate the deployment of BP estimation devices^109^.

The KNN algorithm differs from other ML methods in that it is a nonparametric supervised learning method. In 2019, Chen et al.^110^ compared the measurement performance of five models and reported that the KNN model achieved the best result on the MIMIC II physiological database. In 2021, Fati et al.^77^ embedded a KNN submodel into an automated optimization tool to avoid bias potentially resulting from using a single KNN algorithm. In 2018, Fang et al.^111^ recovered a PPG signal from a remote PPG monitor and leveraged KNN to predict BP parameters.

DT is constructed by choosing a feature in each iteration step to split the sample set best. The metrics used to indicate the “best” split differ on a case-by-case basis and may include information gain. RF algorithms feature the use of multiple DTs. In 2020, Nath and Thapliyal^112^ trained a DT regressor and reported that it did not perform as well as an AdaBoost version. In 2022, Gupta et al.^113^ investigated the prediction accuracy of RF and DT on the UCI and MIMIC I datasets and reported that RF outperformed other models. Farki et al.^114^ developed a clustering-based algorithm to elevate the performance of BP estimation using RF. In 2021, Ma et al.^115^ leveraged the information entropy of signals from wearable devices to train an RF model that had higher accuracy than LR or SVR.

#### DL methods

In CNNs, convolutional kernels are used in the hierarchical architecture of layers to extract spatial and temporal features from data. Although CNN kernels typically have two or more dimensions, CNN kernels used in BP prediction are often one-dimensional because both the input and output signals are temporal sequences. Predictions based on a single modality, such as only PPG, are concise but lack information that could be provided by other modalities. Many researchers have used CNN-based algorithms for multimodal inference using ECG and PPG^116,117^ or ECG and TAG^118^. Other auxiliary techniques, such as squeeze and excitation (SE)^119^, can also improve the performance of CNNs.

RNNs are neural networks that have a “memory” that is consulted while processing each input. Numerous input modalities have been selected for RNNs; researchers have both used a single modality, such as ECG^120,121^ or PPG^122,123^, or a combination of modalities^124–126^. To determine the dynamic significance of each data point in a sequence, the attention mechanism, which is common in the field of natural language processing, has been employed in RNN-based BP estimation methods^120,122^. Typically, information in a neural network is connected between neighboring layers; however, in networks with residual or dense connections, information can “jump” from a node to a node several layers away from it^124^.

Hybrid models that simultaneously leverage the strengths of various learning algorithms have been created and deployed. In particular, CNNs and RNNs^127–131^ are frequently combined because these networks can concurrently extract short- and long-term dependencies. The input modality for hybrid models can be single^128,130–132^ or multiple^127,129,133^. The attention mechanism has also been used in hybrid models to capture the contextual meaning of data points^128,129^. Because individual-level data are often insufficient and DL methods are compute-intensive, finetuning^132^ and transfer learning^134,135^ have been used to increase the performance of methods applied in a new domain. Moreover, physical laws regarding BP or other parameters, such as hemodynamics, can be embedded into the design of an algorithm^136^.

## BP waveform estimation models

In a continuous BP estimation method, the blood pressure waveform is acquired. Because numerous temporally varying details are embedded in a waveform, continuous BP estimation can potentially give doctors more information for diagnosis than beat-to-beat methods can. BP waveform analysis enables inference regarding stroke volume, cardiac output, vascular resistance, and other BP parameters^137^.

### DL-based methods

DL algorithms can fit hidden relationships between input and output data. Thus, DL is applicable in BP estimation for physiological signal inputs extracted from flexible sensors. Due to the versatility of DL algorithms, they can be designed for both beat-to-beat and continuous estimation. In contrast to other nonlearning methods, DL has many intrinsic advantages for continuous BP waveform estimation:

- Almost all functions, regardless of linearity, can be fitted by a well-designed DL algorithm.
- The scale of BP-related medical data is enormous. Compared with analytical models, DL has a greater ability to process data concurrently and is thus suitable for computation-intensive tasks.
- Thanks to the rapid development of algorithmic and computational technologies and the maturity of DL techniques, the ideas and algorithms in this field are amenable to BP waveform prediction.
- DL can automatically extract features from data; nonlearning methods rely on the bespoke features and may inadvertently neglect critical information, resulting in bias.

CNN networks excel at local feature detection and thus are often used for continuous BP estimation. U-Net, a variant of the CNN framework targeted at biomedical image segmentation, has been adapted to ABP estimation with a raw PPG signal as its input^138^. Its adaptation to the one-dimensional time domain, named Wave-U-Net, was used by Cheng et al.^139^ to predict ABP waveforms in 2021. The autoencoder in the network could suppress noise while maintaining the key information. In 2020, Sadrawi et al.^140^ applied a deep convolutional autoencoder that was optimized with a genetic algorithm to estimate BP using PPG. In 2021, Qin et al.^141^ merged a convolution-based deep autoencoder with multidomain adversarial training to learn individual differences between subjects. In 2021, Hill et al.^142^ adapted the V-Net architecture, which was originally designed for image segmentation, for one-dimensional ABP waveform prediction.

In contrast to CNNs, RNNs are frequently used to extract long-term dependencies from data in the time dimension. In 2021, Aguirre et al.^143^ used gated recurrent units (GRUs) to construct an RNN model to assess BP morphology. Harfiya et al.^144^ proposed a long short-term memory (LSTM) model for learning signal-to-signal translation between PPG to ABP. Sideris et al.^145^ tested an LSTM-based RNN and compared it with LR, which is commonly used in beat-to-beat BP estimation.

### Mechanics-based methods

The quantitative relation between BP and the cross-sectional area of the blood vessel allows for the automated tracking of the continuous BP waveform. The dominant modality used for extracting blood vessel diameter is ultrasound. An ultrasound transducer can transform sinusoidal or pulse voltage signals into mechanical ultrasound waves^76,146^. When the ultrasound wave reaches the interfaces between the tissue and the vessel, both transmission and reflection occur. The reflected wave carries critical location information about these interfaces. The ultrasound wave is reflected at both the anterior and posterior interfaces between the tissue and the vessel, enabling changes in vessel diameter over time to be recorded based on the received ultrasound signals^80,147^. The measured diameter change of the vessel can be translated into localized BP waveforms according to the physical model of vessel^148^. BP can be calculated as

7 $$p\left(t\right)={p}_{d}\times {e}^{\alpha \left(A\left(t\right)/{A}_{d}-1\right)}$$

where ${p}_{d}$ is the diastolic pressure, ${A}_{d}$ is the cross-section of the vessel during cardiac diastole, $\alpha$ is the vessel rigidity coefficient, and $A\left(t\right)$ is the cross-section of the vessel over time. If the vessel is assumed to be rotationally symmetrical, $A\left(t\right)$ can be expressed as $\frac{1}{4}\pi {d}^{2}\left(t\right)$, where *d*(*t*) is the measured diameter waveform of the target vessel^70^.

## Calibration

Most of the aforementioned models, in particular the PTT and pulse wave analysis (PWA) based methods, require a calibration process where a few data points or a segment of continuous waveforms are used to fit the model output to the actual BP values obtained from a simultaneously taken invasive or intermittent cuff BP measurement, thus determining the subject-specific constants in the model for consequent BP estimation. Although some mechanics-based approaches^62,70^ are in principle calibration-free, they still require a mapping process to calibrate the local measurement results to the reference BP values obtained from gold standard measurements. Some ML-based algorithms^116,121^ have demonstrated their capability of performing calibration-free BP estimation, however, their adaptability to untrained subject types and long-term stability are yet to be explored.

## Conclusion and future directions

Wearable BP measurement devices have attracted increasing attention from both academia and industry. Several companies (LiveMetric, Healthstats, Sotera, Aktiia, Biobeat, Samsung, etc.) have already commercialized wearable BP solutions and obtained regulatory approval. Yet the realization of accurate and long-term reliable wearable and cuffless BP monitoring systems requires multidisciplinary cooperation. This paper systematically reviewed the devices, signals, algorithms, and other technical issues related to wearable and cuffless BP measurement. Existing sensing technologies cover electrical, optical, and mechanical modalities. Modeling techniques include PTT-based models, ML-based algorithms, and mechanics-based methods. Many challenges and opportunities remain, and future studies may include the following areas:

- It is noted that many cuffless methods with good performances were validated on small size datasets with only healthy subjects or subjects with a relatively narrow age distribution. To ensure generalizability and domain adaptation of the methods, follow-up clinical studies including a broader range of age groups, CV conditions and BP variation patterns are critical. This trend has also been reflected in the recently established IEEE Standard for Wearable Cuffless Blood Pressure Measuring Devices^149^, which sets the requirements of subject selection and BP changes to ensure the devices are adequately exposed to both inter- and intra-individual variations.
- Wearable and cuffless BP monitoring systems should be evaluated under well-established industrial standards to ensure the reliability of the reported measurement performance. The evaluation should include sufficient population size and intra- and inter-individual BP diversity, with a side-by-side comparison of the BP errors between the new and baseline models. In particular, the comparison to generally-accepted baseline models plays a vital role in ML/DL model evaluation because the baseline provides (1) a reasonable performance threshold which the new model should significantly surpass, and (2) reference information of the experimental data for comparison with other studies.
- Snapshot and beat-to-beat BP monitoring evolved to become mature technologies. Continuous BP monitoring, i.e., TAG tracking, is likely to be the next wave of focused research in the field. DL algorithms will be a powerful tool for achieving TAG monitoring.
- A comprehensive understanding of both hardware and software is essential for researching wearable and cuffless BP monitoring. Although BP monitoring can be divided to separate tasks, as illustrated in Fig. 1, an end-to-end design that holistically considers the performance characteristics of each technology component should greatly enhance the efficiency and efficacy of the system.
- Noise is a recurring challenge in BP estimation. Contemporary denoising techniques are effective in the short term. However, maintaining long-term accuracy despite noise is a problem that still requires more feasible solutions.
- Calibration remains necessary to determine the proper modeling parameters but is inconvenient for users. Current calibration methods ensure accuracy but require user-initiate actions, such as measurements using cuff-based oscillometers. Further development of calibration-free BP monitoring methods is desirable.
- The fusion of analytical and ML models is promising. Analytic models provide a physiological base for signal selection and processing, whereas ML methods are more effective in data correlation and prediction. Combining the two approaches can not only enhance the BP estimation accuracy but also provide guidelines on the front-end sensor selection and design.

### Reporting summary

Further information on research design is available in the Nature Research Reporting Summary linked to this article.

## Supplementary information

Reporting Summary

## Data Availability

No new data were generated in this study.
